# Cost-effectiveness analysis of pembrolizumab compared to standard of care as first line treatment for patients with advanced melanoma in Hong Kong

**DOI:** 10.1186/s12962-020-0200-9

**Published:** 2020-01-15

**Authors:** Herbert H. Loong, Carlos K. H. Wong, Linda K. S. Leung, S. C. Tan, Jason Jen, Mary Y. K. Lee, Raquel Aguiar-Ibáñez, Jingshu Wang

**Affiliations:** 1 Department of Clinical Oncology, The Chinese University of Hong Kong, Sha Tin, Hong Kong SAR, China; 2 State Key Laboratory in Translational Research, Hong Kong Cancer Institute, Hong Kong, Hong Kong SAR, China; 3 Department of Family Medicine & Primary Care, The University of Hong Kong, Hong Kong, Hong Kong SAR, China; 4 MSD Asia Pacific, Singapore, Singapore; 5 MSD, Hong Kong, Hong Kong; 6 Merck Sharp & Dohme B.V, Haarlem, The Netherlands; 7 MSD Kenilworth, Kenilworth, NJ USA

## Abstract

**Background:**

Pembrolizumab has been shown to improve overall survival (OS) and progression free survival (PFS) compared to ipilimumab in patients with ipilimumab-naïve advanced melanoma; however, there are no published data on the cost-effectiveness for pembrolizumab compared to standard-of-care treatments currently used in Hong Kong for advanced melanoma.

**Methods:**

A partitioned-survival model based on data from a recent randomized phase 3 study (KEYNOTE-006) and meta-analysis was used to derive time in PFS, OS, and post-progression survival for pembrolizumab and chemotherapy, such as dacarbazine (DTIC), temozolomide (TMZ), and the paclitaxel-carboplatin combination (PC). A combination of clinical trial data, published data, results of meta-analysis, and melanoma registry data was used to extrapolate PFS and OS curves. The base-case time horizon for the model was 30 years with costs and health outcomes discounted at a rate of 5% per year. Individual patient level data on utilities and frequencies of adverse events were obtained from the final analysis of KEYNOTE-006 (cut-off date: 3-Dec-15) for pembrolizumab. Cost data included drug acquisition, treatment administration, adverse event management, and clinical management of advanced melanoma. The distribution of patient weight from the Hong Kong population was applied to calculate the drug costs. Analyses were performed from a payer’s perspective. The incremental cost effectiveness ratio (ICER) expressed as cost in US Dollars (USD) per quality-adjusted life years (QALYs) was the main outcome.

**Results:**

In base-case scenario, the ICER for pembrolizumab as a first-line treatment for advanced melanoma was USD49,232 compared to DTIC, with the ICER values lower than cost-effectiveness threshold in Hong Kong. Results comparing pembrolizumab to TMZ and to PC were similar to that when compared to DTIC. Probability sensitivity analyses showed that 99% of the simulated ICERs were below three times the Gross Domestic Product (GDP) per capita for Hong Kong (currently at $119,274//QALY threshold). In a scenario analysis comparing pembrolizumab with ipilimumab, the estimated ICER was USD8,904.

**Conclusions:**

Pembrolizumab is cost-effective relative to chemotherapy (DTIC, TMZ and PC), and highly-cost-effective compared to ipilimumab, for the first-line treatment of advanced melanoma in Hong Kong.

## Key points

- Although there have been prior publications addressing the cost-effectiveness of checkpoint inhibitors in the treatment of advanced melanomas, most of these prior reports addressed the cost effectiveness between different checkpoint inhibitors (e.g. anti-programmed cell death-1 (PD-1) vs. anti-cytotoxic T-lymphocyte-associated protein 4—CTLA-4) or their use in combination. Cytotoxic chemotherapies are still routinely used as first-line treatment options in various jurisdictions. There remains a paucity of data addressing the cost effectiveness of a checkpoint inhibitor versus cytotoxic chemotherapies.
- We have performed a partitioned-survival model based on data derived from the randomized phase 3 study KEYNOTE-006 in conjunction with prior meta-analyses being used to derive time in PFS, OS and post-progression survival for pembrolizumab as well as chemotherapies.
- A combination of clinical trial data, published data, results from a network meta-analysis and melanoma registry data were used to extrapolate PFS and OS curves. Costing data including drug acquisition and treatment administration were obtained from updated published information by the Hong Kong Hospital Authority, whereas resource utilisation required for the clinical management of adverse events were determined by a team of clinical experts.
- We have concluded that, in Hong Kong, the ICER for pembrolizumab as first-line treatment in advanced melanoma compared with cytotoxic chemotherapies and ipilimumab was USD 49,232 and USD 8904, respectively. Probability sensitivity analyses showed that 99% of simulated ICERs were below three times the Gross Domestic Product (GDP) per capita for Hong Kong (currently at $119,274/QALY threshold).

## Background

Immune checkpoint inhibitors, including the anti- cytotoxic T-lymphocyte-associated protein 4 (CTLA-4) monoclonal antibody ipilimumab, and more recently the availability of the anti-programmed cell death-1 (PD-1) monoclonal antibodies pembrolizumab and nivolumab, have demonstrated significant improvement in treatment outcomes in melanoma. Multiple health regulatory agencies including the United States Food & Drug Administration (FDA) and the European Medicines Agency (EMA) have since approved an expanded indication for pembrolizumab (first line use for patients with advanced melanoma) and the National Comprehensive Cancer Network (NCCN) recommends pembrolizumab as one of the first line treatments for patients with advanced melanoma in its clinical practice guidelines [1]. A paucity of data on the cost-effectiveness of pembrolizumab is available. Wang and colleagues have published a cost effectiveness analysis of pembrolizumab versus ipilimumab in ipilimumab-naïve patients with unresectable or metastatic melanoma from a United States integrated health system perspective [2]. In this scenario, pembrolizumab had higher expected quality adjusted life years (QALYs) and was found to be cost-effective (corresponding incremental cost-effectiveness ratio (ICER) was $81,091 per QALY over a 20-year time horizon) when compared with ipilimumab.

Whilst the above findings are for the United States, it remains to be addressed whether such findings also hold true in other healthcare settings, where there may be fundamental differences in the healthcare funding structure and available alternative treatment options. Moreover, in the prior study, no comparison was made with conventional cyototoxics, which remain the backbone anti-cancer treatments in a large number of jurisdictions, including Hong Kong.

In the base case, we assessed the cost effectiveness of pembrolizumab vs. dacarbazine (DTIC) in patients with advanced melanoma in the first-line setting. As part of sensitivity analyses, two scenarios were further considered, including comparing the cost effectiveness of (i) pembrolizumab versus ipilimumab, and (ii) pembrolizumab versus other cytotoxic chemotherapies (temozolomide—TMZ and paclitaxel–carboplatin combination—PC) in these population, based on the healthcare costs and available therapies in the Hong Kong public healthcare system.

## Methods

**Fig. 1:**
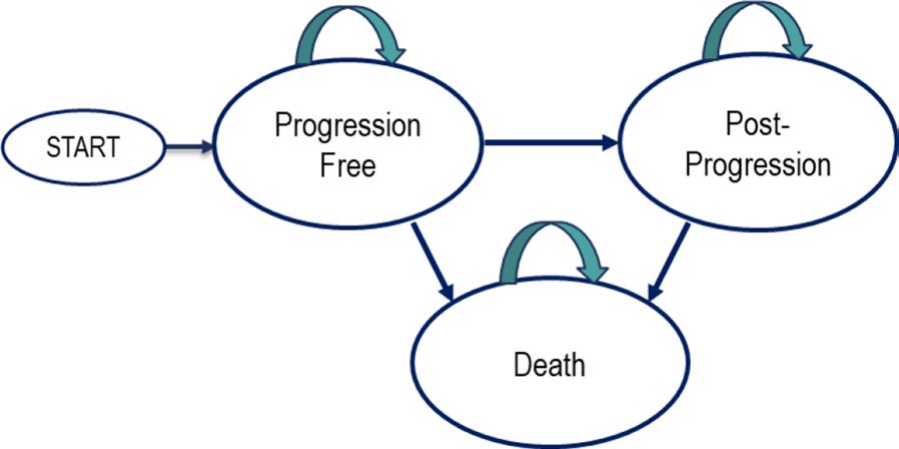
Transition diagram of the simulation model

Using Excel, a partitioned survival model was built with three mutually exclusive health states: progression-free, post-progression, and death. Patients, modeled after those in the KEYNOTE-006 trial, start in the progression-free (PF) state. The progressive disease (PD) state occurs after the first progression defined in the trial by an independent radiologist and oncologist review using the Response Evaluation Criteria in Solid Tumors (RECIST) version 1.1 [3] (Fig. 1).

In the model, pembrolizumab was dosed at 2 mg/kg every 3 weeks (Q3W) and was given for up to 24 months. Those who were still in progression-free survival (PFS) at the end of 24 months were eligible to receive up to 12 months of re-induction treatment if they experienced a disease progression within a 2-year follow-up period.

The time horizon in the base-case analysis of the model was 30 years as it captures the lifetime differences in health benefits and costs of these treatment options. Shorter time horizons were tested in sensitivity analyses.

The modeling of PFS and overall survival (OS) for pembrolizumab (Q3W) was based on data from the KEYNOTE-006 patients treated with pembrolizumab every 3 weeks (i.e. 277 patients in total). Proportions of patients in each health state were calculated based on actual and projected survival curves for PFS and OS. For the PFS curves, Kaplan–Meier estimates from KEYNOTE-006 were used for the first 13 weeks. Week 13 was chosen as the cut-off point to project long-term PFS because there was a discontinuity in the Kaplan–Meier curve related to a protocol-driven radiologic scan. After that, the model used parametric survivor functions fitted to the trial data of week 13 and beyond to project PFS. A Weibull distribution was used for the base case based on the goodness of fit statistics and clinical opinion that the flatter tail would better reflect the long-term benefit of immunotherapy.

For the OS curves, the model used the Kaplan–Meier estimates of pembrolizumab from KEYNOTE-006 for the first 100 weeks. Beyond 100 weeks, a long-term ipilimumab study by Schadendorf et al. [4] was utilized in order to capture the plateau in the OS curve found in immunotherapy. Specifically, a hazard ratio function of pembrolizumab vs. ipilimumab estimated from the KEYNOTE-006 trial was applied to the aforementioned long-term ipilimumab study to derive the OS curve for pembrolizumab until week 156, beyond which the hazard rates from the American Joint Committee on Cancer (AJCC) melanoma registry were used to estimate OS in the pembrolizumab arm [5]. Since the melanoma registry only reported cancer related deaths [6], age-specific background mortality rates were additionally incorporated in the model, derived from 2017 Hong Kong life tables and using a weighted average of male and female mortality risks (which reflected the gender distribution of participants in the KEYNOTE-006 trial). was also incorporated because there is currently no randomized clinical trial comparing the survival benefit of pembrolizumab versus DTIC. Therefore, PFS and OS curves for DTIC were obtained by applying a constant hazard ratio to the curves of pembrolizumab, estimated from a Bayesian network meta-analysis that allowed an indirect comparison between pembrolizumab and DTIC using a second-order proportional hazards model (since there was insufficient evidence against proportional hazards between pembrolizumab and dacarbazine over time) [7]. Similarly to what was used for the OS pembrolizumab curve, the hazard rates from the AJCC melanoma registry were used beyond 156 weeks to estimate OS for patients in the DTIC arm, incorporating as well background mortality derived from 2017 Hong Kong life tables to capture non-cancer related deaths [5].

The model assumed that best supportive care (included ‘no active treatment’) was the only subsequent therapy administered after progression for both treatment drugs. This assumption was applied because the trial data did not show a significant difference in post-progression drug use between the two arms and other assumptions would require speculation regarding efficacy from various sequences and durations of drug use.

Quality of life and adverse event data were mainly derived from the final analysis of the ongoing KEYNOTE-006 trial [8] and other published sources [9]. The costings utilized in the analysis were extracted from various published sources reported in 2018 [10] and were described below.

### Costs

Costs were estimated from a payer’s perspective. The study used listed drug prices as made available by the Hong Kong Hospital Authority [10]. Pembrolizumab was $2564.10 per 100-mg vial and DTIC was $56 per 100 mg. For each administration, pembrolizumab was dosed at 2 mg/kg and DTIC at a dose of 1000 mg/m^2^. Drug acquisition costs were calculated in whole vials rounded up at the patient level. Based on the patient weight distribution from Hong Kong local data (average body weight = 65 kg), the average number of vials was 1.84 of 100-mg for pembrolizumab and 10 of 100-mg for DTIC per administration. DTIC patients took the drug every 3 weeks unless the drug was stopped due to unacceptable toxicity or disease progression. The model projected the average number of doses of DTIC per patient as 11.0. Pembrolizumab patients in the trial took the drug every 3 weeks until disease progression, the onset of unacceptable side effects, an investigator’s decision to discontinue treatment, withdrawal or patient consent, or 24 months of therapy. Patients who were still progression-free at the end of 24 months were eligible to receive up to 12 months of re-induction treatment if they experienced a disease progression within a 2-year follow-up period. The model projected that 41% of the patients in PFS would receive re-induction. The projected drug duration over 30 years across all pembrolizumab patients was 12.7 months (where it was observed to be 9.6 months at the database-lock time of trial), and the projected average number of doses of pembrolizumab per patient was 19.4.

Drug administration costs per dose were estimated from rates extracted from local Hong Kong data. The model also incorporated costs for routine oncology office visits, lab tests, scans and other resources used in the different health states based on the INTUITION study (see Table 1) [11]. The model also included a one-time cost of terminal care to approximate health care cost in the last 6 months of life as estimated in Wong et al. 2007 [12].

**Table 1:** Model inputs

	Intervention	Base case comparator	Additional comparators tested in scenario analyses		
	Pembrolizumab	DTIC	Ipilimumab	Temozolomide	Paclitaxel/carboplatin
Survival extrapolation for progression free survival (PFS) and overall survival (OS)					
PFS funtional form^a^	Weibull	Constant HR from NWMA	Log-normal	Assumed the same as for DTIC	Assumed the same as for DTIC
OS functional form^b^	Lognormal model for HR	Constant HR from NWMA followed by registry data	KEYNOTE-006 ipilimumab arm, long-term ipilimumab data from Schadendorf et al. [4] and AJCC data.	Assumed the same as for DTIC	Assumed the same as for DTIC
Utility: mean (95% confidence interval)					
Utility: ≥ 360 days till death	0.82 (0.81, 0.83)				
Utility [270,360) days till death	0.74 (0.69, 0.79)				
Utility [90, 270) days till death	0.69 (0.65, 0.73)				
Utility [30,90) days till death	0.60 (0.54, 0.66)				
Utility < 30 days till death	0.42 (0.29, 0.56)				
Utility for PFS	0.82 (0.81, 0.83)	0.79 (0.77, 0.80)	0.82 (0.81, 0.83)		
Utility for post-progression	0.72 (0.70, 0.74)				
Adverse events (AE)					
Colitis, grade 3 and above^f^	1.8%	0.0%	6.3%	0.0%	0.0%
Diarrhea (excl. colitis), grade 2 and above	3.6%	0.0%	7.8%	0.0%	0.0%
Endocrine disorders, any grade	12.3%	0.0%	5.5%	3.0%	0.0%
Neutropenia, grade 3 and above	0.0%	11.9%	0.0%	3.0%	18.8%
Thrombocytopenia	0.0%	5.1%	0.0%	7.0%	
Hemorrhage (non-CNS/pulmonary)	0.0%	0.0%	0.0%		5.8%
Asthenia	0.0%	0.0%	0.0%	3.0%	0.0%
Headache	0.0%	0.0%	0.0%	6.0%	0.0%
Pain	0.0%	0.0%	0.0%	7.0%	0.0%
Constipation	0.0%	0.0%	0.0%	3.0%	0.0%
Nausea	0.0%	0.0%	0.0%	4.0%	0.0%
Vomiting	0.0%	0.0%	0.0%	5.0%	0.0%
# of treatment for endocrine disorders	3.23	0	3.00	0	0
Costs of AE management	$863	$92	$1196	$672	$767
Disutility of an AE^e^	0.15 over 8 weeks				
Drug costs					
Unit cost of drug	$2564 per 100 mg vial	$56 per 100 mg vial	$ 5.897 per 50 mg vial	$14.28 per 20 mg vial	Paclitaxel: $25.77 per 100 mg Carboplatin: $30.69 per 450 mg
Dose per administration	200 mg Q3W	1000 mg/m^2^	3 mg/kg Q3W for a maximum of 4 doses	1000 mg/m^2^	Paclitaxel: 300 mg Carboplatin: 525 mg
Mean number of vials per 3 weeks (based on whole vials at the patient level)	1.84 vials	17.5	4,40 of 50 mg vials	1750 mg (1000 mg/m^2^)	Paclitaxel: 3 of 100 mg vials Carboplatin: 1.17 of 450 mg vials
Mean cost of drug administration^c^	$91.67 per administration	$91.67 per administration	$91.67 per administration	$0 (oral drug)	$91.67 per administration
Total drug cost for each dose	$4706	$980	$25.937	$1.249,50	Paclitaxel: $77.31 Carboplatin: $61.38 Total: $138.69
Disease management costs^d^					
Management during PFS	$144/week				
Management during post progression	$109/week				
Death related costs	$24,089 (last 6 months of life)				

^a^Functions selected based on Akaike Information Criterion and Bayesian Information Criterion for best fit in weeks 13 and beyond from the trial data

^b^Functions selected based on Akaike Information Criterion and Bayesian Information Criterion for best fit from the trial data

^c^Drug administration costs are from local Hong Kong data. DTIC and pembrolizumab are given once in every 3 weeks until disease progression or 24 months and 41% of patients in PFS at the end of 2 years are projected to receive a second course for a maximum of 12 months

^d^Disease management costs include oncology office visits, lab tests, scans and other resources which are enlisted in Appendix: Table 4 and the costs are based on Hospital Authority itemized charges as of July 2013

^e^AE costs were based on frequencies of grade 3 or higher AEs that impacted at least 3% of patients in either arm and the costs were extracted from the Hospital Authority Ordinance [14]. AE disutility was measured by pooling utility scores in patients experiencing an AE versus patients in weeks without an AE and AEs were then modeled as lasting 8 weeks. Costs for managing colitis and diarrhea were $11,785 and $2892 respectively. A cost of $1379 was incurred for treating endocrine disorders every 6 months while Neutropenia of grade 3 or above was associated with a cost of $779 [14]. Even though thrombocytopenia was also a prominent adverse event, its management cost was in significant and hence assumed to be $0

^f^An exception to the 3% rule was applied for this AE since it had a 6.3% incidence with Ipilimumab and it was the grade 3–4 AE with the highest incidence in the pembrolizumab Q3W group

Adverse events (AE) of severity grade 3 to 5 that impacted at least 3% of the patients in at least one of the treatment arms were included in the model for both arms [8, 9]. In addition, grade 2+ diarrhea was included due to its economic impact. Costs of endocrine disorders are assumed to be incurred once every 6 months [13]. The costs of managing the included grade 3–5 AE were taken from Gazetted prices from the Hospital Authority Ordinance (Chapter 113) [14].

### Utility scores

Utility scores were based on quality of life data collected in KEYNOTE-006 trial with missing values excluded. The European Quality of Life Five Dimensions Questionnaire (EuroQoL EQ-5D)—was administered at certain visits to pembrolizumab and ipilimumab patients. It was also administered at drug discontinuation visits and day 30 safety follow-up visits. Responses to the EQ-5D questionnaire were converted to population-based utility values using a mixed algorithm (where US-based scores were applied to US patients, UK-based scores for UK patients and EU-based scores for all other patients were used) as the Hong Kong specific algorithm is not currently available. Mean EQ-5D utility scores associated with the following time-to-death categories were calculated: 360 days or more, 270–360 days, 90–270 days, 30–90 days and under 30 days. Death was assigned a utility of 0 [2].

Utility scores associated with patients experiencing grade 3–5 adverse events were also compared with those when patients were not experiencing adverse events. The difference between visits with and without grade 3–5 adverse events were used to estimate the average disutility associated with adverse events. For the base case analysis, the mean utility decrement for an AE from the pooled data analysis (i.e. 0.15) was used, and the duration of the AEs was taken to be 8 weeks. For each health state, a specific cost and quality-of-life adjustment weight was assigned for each 1-week cycle to calculate cumulative costs and cumulative QALYs over the model time horizon.

AE-related costs and utility decrements were applied separately to each drug assuming the events occurred at the beginning of the study. Costs and QALYs were discounted at a rate of 5% per year. To conduct the cost-effectiveness assessment, the model was used to project costs, life years, QALYs, and the incremental cost per QALY gained associated with using pembrolizumab versus comparators in treatment-naïve patients.

### Sensitivity analyses

Sensitivity analyses conducted included: scenario analyses, deterministic one-way sensitivity analyses and probabilistic sensitivity analyses.

The scenario sensitivity analyses examined the impact of alternative comparators (not currently reimbursed or used as first line therapies in Hong Kong), several different methodologies used in the extrapolation of the survival (PFS and OS) curves, utility estimates based on progression-based health states (i.e. for each health state before and after progression). different time horizons, discount rates, and different assumptions regarding the treatment strategy for pembrolizumab.

Scenario sensitivity analyses considering alternative comparators not currently reimbursed or not used as first line treatments for advanced melanoma in Hong Kong included comparisons of pembrolizumab versus the immunotherapy drug, ipilimumab, as well as other chemotherapy drugs such as TMZ and the PC combination. For ipilimumab, a similar modelling approach was used to that of pembrolizumab, with clinical efficacy and safety mainly derived from patients in the KEYNOTE-006 ipilimumab arm, and long-term ipilimumab data from Schadendorf et al. [4] and AJCC data. For other chemotherapy drugs, the same clinical efficacy as that for DTIC was assumed since indirect treatment comparisons were only possible for pembrolizumab versus DTIC. Based on clinical opinion, the efficacy across different chemotherapies was expected to be similar. This assumption is supported by studies showing that chemotherapies are unlikely to have survival benefit in terms of tumor response and improved time to progression, or improved overall survival over best supportive care (BSC) in advanced melanoma patients [15–17]. Additionally, there are no randomised controlled trials demonstrating an improvement in survival with DTIC relative to BSC.

The list price for ipilimumab was $5897.43 per 50 mg vial, and it was administered at a dose of 3 mg/kg. Based on the patient weight distribution from Hong Kong local data, an average of 4.40 vials of 50 mg were required. The list price of TMZ was $0.71 per mg and with a dosage of 1000 mg/m^2^ (200 mg/m^2^ five times a week), the cost per dose of TMZ was estimated to be $1249.50. Paclitaxel and carboplatin were available at the list prices of $0.26 per mg and $0.07 per mg, respectively with their respective doses of 175 mg/m^2^ and 300 mg/m^2^ given once every 3 weeks (average body surface area = 1.75 m^2^). The estimated costs of these alternative comparators considered in the scenario analyses are presented in Table 1.

Deterministic one-way sensitivity analyses focused on varying model parameter values related to the base case comparison of DTIC with pembrolizumab. Parameters values were varied for the extrapolation functions across the estimated 95% confidence intervals, utilities were modified by plus or minus 20%, disease management costs by 25%, and AE management costs from 50 to 200% as best guesses for their potential range given limited available quantitative data.

Furthermore, a probabilistic sensitivity analysis (PSA) was run based on 1000 sets of simultaneous samples from specified probability distributions of the model inputs. The underlying distributions included a beta distribution using the mean and standard error for the utilities based on the clinical trial, and log normal distributions for the cost inputs using means equal to the base case value and standard errors as reported in the literature, or set conservatively at 20% of the base case value. In addition, the PSA incorporated uncertainty in the functional forms of the extrapolation functions based on assigning distributions for the key parameters characterizing the extrapolation functions. Based on the 1000 estimates for the incremental cost per QALY gained, the probability of pembrolizumab and comparator being cost-effective at various willingness-to-pay thresholds were displayed using a cost-effectiveness acceptability curve.

## Results

### Base-case analysis

Detailed results from the base-case analyses are shown in Table 2 and Fig. 1. Patients treated with pembrolizumab spent an average of 2.36 years in the progression-free health state and 5.48 years in the post-progressive health state, resulting in a mean survival estimate of 7.83 years. In the DTIC arm, patients spent an average of 0.61 years in the progression-free health state and 2.13 years in the PD state for a mean survival time of 2.74 years. Hence, pembrolizumab was associated with a gain in mean survival of 5.09 years.

**Table 2:** Base case results for deterministic and probabilistic analyses: pembrolizumab vs DTIC

Model Estimates	DTIC	Pembrolizumab	Difference
Deterministic sensitivity analysis: mean			
Effectiveness (not discounted)			
Progression-free life years	0.61	2.36	1.75
Post progression life years	2.13	5.48	3.34
Life years	2.74	7.83	5.09
QALYs	1.64	6.3	4.7
Effectiveness discounted			
QALYs	1.64	4.28	2.64
Costs discounted, $			
Medication costs	10,249	86,937	76,688
Drug administration costs	959	1693	735
AE costs	92	863	771
Additional costs of care	34,779	51,638	16,859
Total	46,079	141,131	95,052
Cost effectiveness, $			
Incremental cost per LY gained			18,668
Incremental cost per QALY gained			35,993
Probabilistic sensitivity analysis: mean (95% class interval)			
Effectiveness discounted			
LYs	2.77 (1.42, 4.41)	7.82 (7.40, 8.21)	5.05 (3.37, 6.49)
QALYs	1.65 (0.86, 2.59)	4.27 (4.06, 4.48)	2.62 (1.66, 3.46)
Costs discounted, $			
Total costs	47,124 (35,410, 62,214)	140,612 (127,407, 156,590)	93,488 (80,879, 105,870)
Cost Effectiveness, $			
Incremental cost per LY gained			18,510 (14,450, 26,412)
Incremental cost per QALY gained			35,681 (27,509, 53,489)

**Fig. 2:**
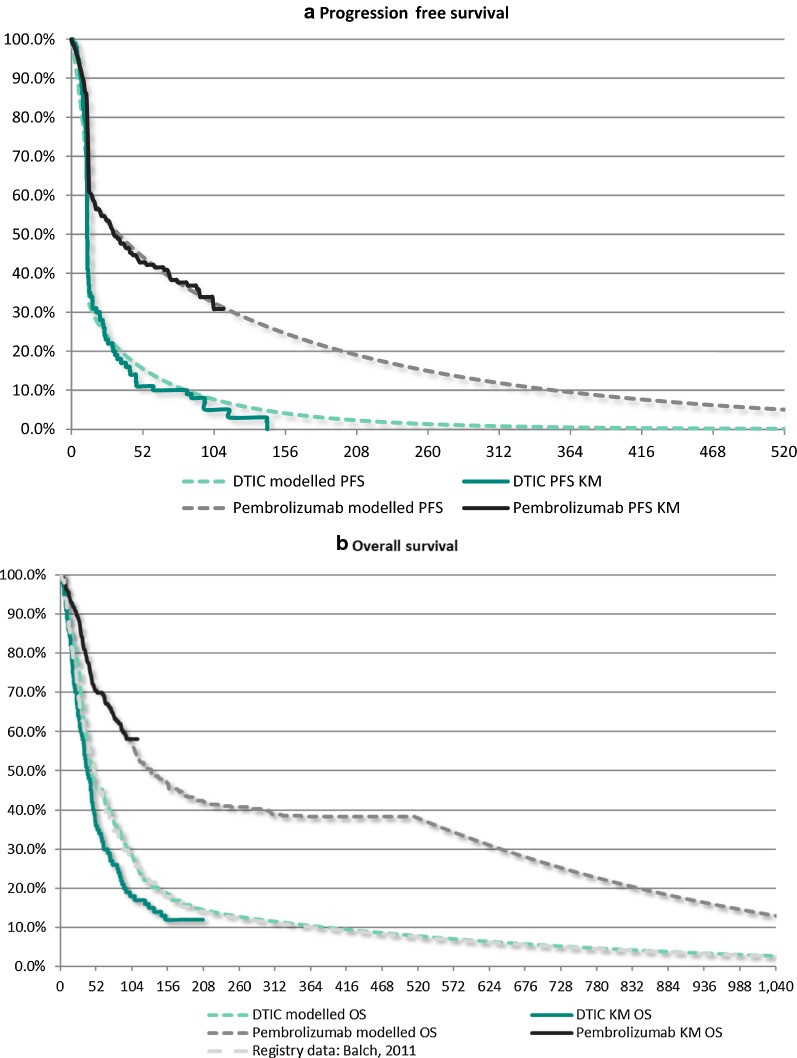
Modelled progression-free and overall survival for pembrolizumab and DTIC (time in weeks)

In terms of QALYs, pembrolizumab was associated with an average (discounted) gain of 2.64 QALYs over DTIC. In addition, the base-case model projected a difference of $95,052 in the total average per-patient direct cost of treatment with pembrolizumab versus DTIC. Therefore, the ICER for pembrolizumab was $35,993/QALY ($18,668 per LY) over a 30-year time horizon (Fig. 2).

### Scenario sensitivity analyses

Detailed results of the scenario sensitivity analyses are presented in Table 3.

**Table 3:** Results of the scenario sensitivity analyses

	Comparator^a^			Pembrolizumab			Pembrolizumab vs. comparator				
	LYs	QALYs	Costs	LYs	QALYs	Costs	∆ LYs	∆ QALYs	∆ cost	ICER (∆cost/LY)	ICER (∆cost/QALY)
Base case	2.74	1.64	46,079	7.83	4.28	141,131	5.09	2.64	95,052	18,668	35,993
Alternative comparators not reflecting clinical practice											
Pembrolizumab vs. ipilimumab	5.84	3.22	130,793	7.83	4.28	141,131	1.99	1.06	10,338	5196	9761
Pembrolizumab vs. temozolomide	2.74	1.63	45,409	7.83	4.28	141,131	5.09	2.65	95,722	18,800	36,169
Pembrolizumab vs. paclitaxel/carboplatin	2.74	1.64	36,551	7.83	4.28	141,131	5.09	2.64	104,580	20,540	39,574
Impact of changing the method of survival (PFS, OS) extrapolation											
PFS modelled based on log-logistic distribution	2.74	1.64	47,593	7.83	4.28	140,423	5.09	2.64	92,830	18,232	35,152
PFS modelled based on log-normal distribution	2.74	1.64	48,496	7.83	4.28	139,845	5.09	2.64	91,349	17,941	34,591
OS modelled based on generalised-gamma HR function	2.66	1.60	45,844	7.58	4.15	140,458	4.92	2.56	94,614	19,236	36,967
Varying utility estimates											
Progression-based utilities	2.74	1.58	46,079	7.83	4.04	141,131	5.09	2.46	95,052	18,668	38,642
Varying time horizons											
5 years	1.62	1.13	40,472	2.85	2.04	120,333	1.23	0.91	79,861	64,912	87,333
10 years	2.12	1.41	43,652	4.79	3.14	129,090	2.67	1.74	85,438	32,041	49,218
20 years	2.60	1.60	45,672	7.14	4.10	139,111	4.54	2.50	93,438	20,595	37,367
Varying discount rates											
0% for both health benefits and costs	2.74	2.12	52,624	7.83	6.29	166,075	5.09	4.18	113,452	22,282	27,153
0% for health benefits, 7% for costs	2.74	2.12	44,230	7.83	6.29	134,483	5.09	4.18	90,253	17,726	21,601
7% for health benefits, 0% for costs	1.99	1.51	52,624	4.72	3.78	166,075	2.73	2.27	113,452	41,482	50,074
7% for both health benefits and costs	1.99	1.51	44,230	4.72	3.78	134,483	2.73	2.27	90,253	32,999	39,835
Varying practice patterns											
Treatment until progression	2.74	1.64	46,079	7.83	4.28	216,013	5.09	2.64	169,934	33,376	64,349
0% of patients who completed the treatment course received second treatment course	2.74	1.64	46,079	7.83	4.28	132,354	5.09	2.64	86,275	16,945	32,670
100% of patients who completed the treatment course received second treatment course	2.74	1.64	46,079	7.83	4.28	153,653	5.09	2.64	107,574	21,128	40,735

^a^DTIC represents the base case comparator reflecting clinical practice unless otherwise specified

#### Comparison of pembrolizumab with ipilimumab

Pembrolizumab was compared with ipilimumab in the first line setting. Patients treated with ipilimumab spent an average of 0.81 years in the PFS and 5.03 years in the PD state resulting in a total survival of 5.84 years. Pembrolizumab improved the mean survival by 1.99 years with most of the gain in PFS. Pembrolizumab was also associated with a QALY gain of 1.06 over ipilimumab. The analysis results indicated a difference of $10,338 in the total average per-patient direct cost of treatment with pembrolizumab versus ipilimumab. The incremental cost per QALY gained was $9761/QALY over a 30-year time horizon.

#### Comparison of pembrolizumab with temozolomide

Pembrolizumab was also compared with the chemotherapy drug, TMZ in the first line setting. Patients treated with TMZ spent an average of 0.61 year in the PFS and 2.13 years in the PD state resulting in a total survival of 2.74 years. Pembrolizumab improved the mean survival by 5.09 years. Pembrolizumab was also associated with a QALY gain of 2.65 over TMZ. The analysis results indicated a difference of $95,722 in the total average per-patient direct cost of treatment with pembrolizumab versus TMZ. The incremental cost per QALY gained with pembrolizumab vs. TMZ was $36,169/QALY over a 30-year time horizon.

#### Comparison of pembrolizumab with paclitaxel/carboplatin

Pembrolizumab was compared with the PC combination in the first line setting. Patients treated with PC spent an average of 0.61 year in the PFS and 2.13 years in the PD state resulting in a total survival of 2.74 years. Pembrolizumab improved the mean survival by 5.09 years. Pembrolizumab was also associated with a QALY gain of 2.64 over PC. The analysis results indicated a difference of $104,580 in the total average per-patient direct cost of treatment with pembrolizumab versus PC. The incremental cost per QALY gained with pembrolizumab vs. PC was $39,574/QALY over a 30-year time horizon.

#### Impact of changing the method of survival (PFS, OS) extrapolation

When a log-logistic and a log–normal function, instead of a Weibull, was used for the PFS of pembrolizumab treated patients, the ICER was reduced to $35,152/QALY and $34,591/QALY respectively.

Using generalized-gamma to model OS for pembrolizumab (instead of a log-normal parametric function, as for the base case), the life expectancy over a 30-year time horizon was projected to be 7.58 years (undiscounted), and the ICER was $36,967 per QALY (discounted).

#### Varying utility estimates

Instead of categorizing health states for patients’ quality of life by their time to death, utilities can also be estimated on the basis of the patients’ progression status (before and after progression). When utility values were assigned to each health state using trial data by treatment arm for PFS and pooled data for PD state (and assigning a utility of 0 to death), the cost-effectiveness result was $38,642/QALY.

#### Varying time horizons

The time horizon was varied from 5 years to 20 years which resulted in the ICER varying from $87,333 to $37,367. In general, shorter time horizons resulted in higher ICERs as most of the treatment costs were incurred in the first 2 years of the time horizon but survival gains continued to be realized after 2 years.

#### Varying discount rates

The discount rates for both costs and health outcomes were varied from 0 to 7%. The ICER ranged from $21,601 in the most advantageous situation where the costs were discounted at 7% and the health benefits at 0% to $50,074 where costs were discounted at 0% and health benefits at 7%.

#### Varying practice patterns

Treating pembrolizumab patients until disease progression instead of following the KEYNOTE-006 protocol has a substantial impact on the ICER. Specifically, the ICER would increase from $35,993 (per the KEYNOTE-006 protocol) to $64,349/QALY if all patients are treated until progression, where only treatment costs are varied but no benefit is assumed in delaying PFS or OS when treating patients for a longer time. The ICER was also affected by the assumed proportion of patients receiving a second (12 month maximum) course of treatment. When the proportion of patients with complete response, partial response, or stable disease at the end of 24 months of treatment who would receive a second course of treatment was varied from 0 to 100%, the ICER results ranged from $32,670 per QALY to $40,735 per QALY.

### Deterministic sensitivity analyses

**Fig. 3:**
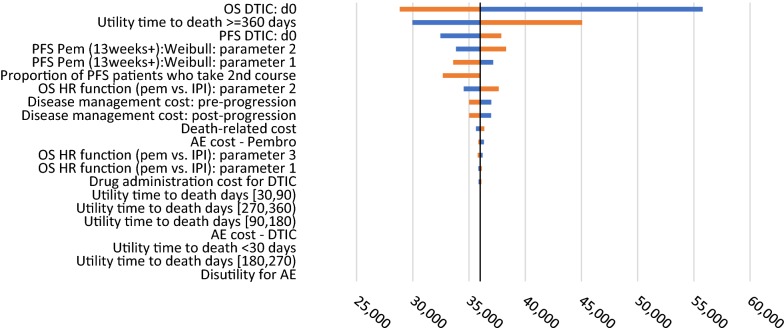
Tornado Diagram for the ICER of pembrolizumab vs. DTIC. (a) The vertical line in the middle indicates the ICER of the base-case scenario ($35,993). (b) The orange bar indicates the ICER result when the minimum value of the input is used, while the blue bar indicates the ICER result when that maximum value of the input is used

**Fig. 4:**
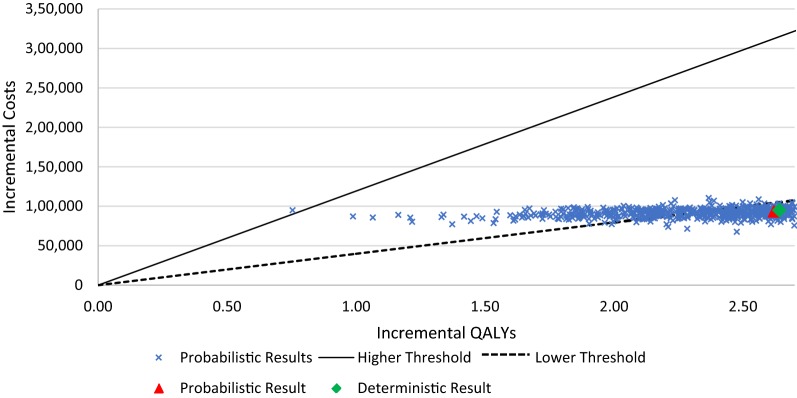
Cost-effectiveness plane for pembrolizumab vs. DTIC. Higher threshold = 3 * GDP per capita; Lower threshold = 1 * GDP per capita

Figure 3 shows the impact of parameter variation on the ICER as derived from the deterministic sensitivity analysis. Model inputs that had the greatest impact on the projected ICER were the parameters in the OS and PFS survivor functions, and utility within the time period more than 1 year away from death. It should be noted that the sensitivity analyses related to the parameters of the survival functions are meant to indicate whether the PFS and OS data are impactful, but are unlikely to reflect the actual potential ranges of the PFS and OS results. Across all the scenarios used in the sets of one-way deterministic sensitivity analyses, the ICER ranged from $28,833/QALY to $55,791/QALY (Fig. 4).

### Probabilistic sensitivity analyses

**Fig. 5:**
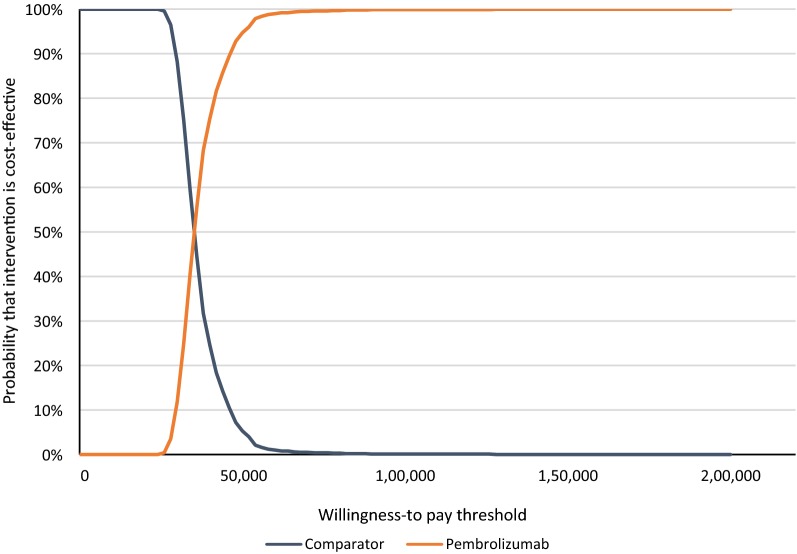
Cost-effectiveness acceptability curve for pembrolizumab vs. DTIC

Results of the Monte Carlo simulations to estimate the incremental cost per QALY gained are summarized as a cost-effectiveness plane and a cost effectiveness acceptability curve in Fig. 5, respectively. Based on the latter curve, the probability of pembrolizumab being cost-effective versus DTIC at willingness-to-pay thresholds of $100,000/QALY, $76,000/QALY, and $50,000/QALY were, respectively, 100%, 100% and 95%.

## Discussion

Understanding the impact of treatment decisions on costs and outcomes can help decision makers, particularly those in large integrated health systems, improve efficiency and promote value-based treatment choices. Building on the most recently available data, this study provides the first evaluation of the cost effectiveness of pembrolizumab versus DTIC as a treatment for treatment-naïve patients with advanced melanoma. Specifically, the model projected a gain of 2.64 discounted QALYs at an incremental, discounted per-patient costs of $95,052 with pembrolizumab versus DTIC, with a corresponding ICER of $ 35,993/QALY over a 30-year time horizon. The ICER is much lower than the WHO threshold of three times the Gross Domestic Product (GDP) per capita for Hong Kong, which is currently at $119,274/QALY. The results suggest that pembrolizumab is a cost effective first-line treatment option for patients with advanced melanoma in Hong Kong.

The strengths of the model include the use of the clinical trial data and the best available methods and literature data for extrapolating survival beyond the trial. In addition, the model incorporates published real world data on relevant cost inputs and EQ-5D-based estimates of utilities for the relevant health states taken directly from the KEYNOTE-006 trial.

The results of the model were robust to a variety of sensitivity analyses, including deterministic and probabilistic sensitivity analyses as well as different scenarios and several variations in the methods employed for extrapolating survival past the trial period.

## Limitations

The study only included direct medical costs to reflect a Hong Kong payer’s perspective. Therefore, other direct non medical costs such as transportation or societal costs (lost productivity or caregiver costs) were not included in this analysis. Exclusion of these cost may underestimate the overall benefits of pembrolizumab. Local data on clinical efficacy and safety were not available which could have contributed to the OS and PFS specific to Hong Kong advanced melanoma patients. Much of the data was from the ongoing KEYNOTE-006 trial with a median follow-up of 23 months, and data was not available on the re-induction of pembrolizumab at disease progression per the KEYNOTE-006 protocol [8, 18]. It is unclear what percentage of patients could benefit from this strategy and what the benefit would be. The model projected that 41% of the pembrolizumab patients who are in complete response, partial response, or having stable disease would receive an assumed 12-month course of treatment after 2 years, and the model was sensitive to this assumption.

There is also uncertainty regarding whether treatment with pembrolizumab would continue until disease progression. The protocol stipulates that patients remaining progression-free state for 2 years must stop treatment after a 2-year treatment course and then re-initiate if there is progression within 2 years [8]. However, patients in clinical practice may not follow the protocol and instead opt to stay on treatment indefinitely. Over a 30-year time horizon, assuming all the patients stay on pembrolizumab until disease progression results in a significantly higher ICER. In clinical practice, treatment patterns may also differ from those in the trial. However, little data is available to indicate how many patients would stay on treatment past 2 years.

Drug duration was modeled as time to progression using RECIST 1.1 criteria. However, immune related response criteria (irRC) may be more relevant [19]. Notably, a recent study by Hodi et al. using RECIST vs. irRC found that RECIST may underestimate the benefit in roughly 15% of patients in terms of better quality of life and lower disease management costs in the progression-free state [19]. Using irRC may also mean longer drug duration. Nonetheless, with a 24-month cap, this is unlikely to meaningfully impact the cost-effectiveness results.

The results are sensitive to long-term survival results, which at this point have to be extrapolated as limited data is available on the long-term survival of pembrolizumab patients. Although rigorous methodological approaches have been applied and several sensitivity analyses were performed to assess potential variation in the results, actual survival patterns may be different.

The model assumed that best supportive care was the only subsequent-line of therapy administered to patients after progression. More data is needed to see the pattern of post-progression drug use, which could affect both the costs and outcomes post progression and thus the ICER. However, this was beyond the scope of the original research question, which is the comparison of pembrolizumab and another active treatment, all else equal.

Base-case utilities were based on a time to death approach. Sensitivity analyses were presented considering progression-based utilities, for which the corresponding values may not fully capture patients’ quality of life during the entire post-progression phase as they were generally collected shortly after progression.

Finally, a partitioned survival model using a piece-wise approach was implemented for the purpose of this cost-effectiveness assessment. Alternative, more flexible modelling approaches, such as cure models, were not further evaluated in our study. Cure models can be useful in situations where the studied population is a combination of cured patients (long term survivors who are expected to never experience the event) and uncured patients (i.e. patients susceptible of experiencing the event of interest). Lack of longer term data, needed to justify the cure threshold assumed for long term survivors, precluded us from considering the use of a cure model for this analysis. Further research is guaranted to evaluate the impact of using alternative, more flexible approaches when modelling the cost-effectiveness of pembrolizumab as a first line treatment for patients with advanced melanoma in the presence of longer term follow-up data.

## Conclusion

The KEYNOTE-006 trial established the clinical benefit of pembrolizumab as a new standard of care for melanoma patients. The model developed here indicated that pembrolizumab was likely to be a cost-effective option from the perspective of a Hong Kong integrated health system over a 13-year time horizon. Further research is needed to confirm the long-term costs and benefits, and thus the cost-effectiveness, of pembrolizumab compared to standard of care.

## Data Availability

Raw data and material used in this study and preparation of this manuscript can be obtained from the author for review.

## Appendix

See Table 4.

**Table 4:** Unit costs

Type of health care resource	Unit cost (USD)	Resource utilization	Total cost (unit cost × resource utilization)
Progression free state management cost			
Inpatient admissions			
Time in hospital (days)	$600.00	0.93	$55.80
Time in ICU (days)	$2949	0.003	$8.85
Outpatient admissions			
Hospital visits	$142.31	0.014	$1.99
Office-based visits	$49.36	0.011	$0.54
Radiation			
Radiotherapy sessions	$496.15	0.017	$8.43
Labs			
Hematology	$49.36	0.092	$4.54
Chemistry—n (%)	$116.03	0.094	$10.91
Thyroid function—n (%)	$79.49	0.037	$2.94
ACTH simulation test—n (%)	$311.34	0.008	$2.49
Imaging			
X-ray	$24.36	0.003	$0.07
PET/CT scan	$1339.74	0.025	$33.49
MRI	$1000.00	0.013	$13.00
Ultrasonography	$256.41	0.004	$1.03
Total cost			$144.09
Progressive disease state management cost			
Inpatient admissions			
Time in hospital (days)	$600.00	0.078	$46.80
Outpatient admissions			
Hospital visits	$142.31	0.002	$0.28
Office-based visits	$49.36	0.005	$0.25
Emergency care visits	$126.92	0.001	$0.13
Radiation			
Radiotherapy sessions	$496.15	0.019	$9.43
Labs			
Hematology	$49.36	0.038	$1.88
Chemistry—n (%)	$116.03	0.033	$3.83
Thyroid function—n (%)	$79.49	0.021	$1.67
ACTH simulation test—n (%)	$311.34	0.001	$0.31
Imaging			
X-ray	$24.36	0.001	$0.02
PET/CT scan	$1339.74	0.023	$30.81
MRI	$1000.00	0.012	$12.00
Ultrasonography	$256.41	0.007	$1.79
Total cost			$109.20
